# The Genomic Regions That Contain Ochratoxin A Biosynthetic Genes Widely Differ in *Aspergillus* Section *Circumdati* Species

**DOI:** 10.3390/toxins12120754

**Published:** 2020-11-29

**Authors:** Jéssica Gil-Serna, Covadonga Vázquez, Belén Patiño

**Affiliations:** Department of Genetics, Physiology, and Microbiology, Faculty of Biology, University Complutense of Madrid, Jose Antonio Nováis 12, 28040 Madrid, Spain; covi@ucm.es (C.V.); belenp@ucm.es (B.P.)

**Keywords:** *Aspergillus ochraceus*, *Aspergillus steynii*, *Aspergillus westerdijkiae*, biosynthetic cluster, ochratoxin A

## Abstract

*Aspergillus* section *Circumdati* includes 27 species, some of which are considered ochratoxin A (OTA) producers. However, there is considerable controversy about their potential OTA synthesis ability. In this work, the complete genomes of 13 species of *Aspergillus* section *Circumdati* were analyzed in order to study the cluster of OTA biosynthetic genes and the region was compared to those previously reported in *A. steynii* and *A. westerdijkiae*. The results obtained reveal that the genomes of some species in this section, including *A. affinis*, *A. cretensis*, *A. elegans*, *A. muricatus*, *A. pulvericola*, *A. roseoglobulosus*, and *A. subramanianii*, contain a potentially functional OTA biosynthetic cluster. Therefore, they might be able to synthesize the toxin. On the contrary, *A. melleus*, *A. ochraceus*, *A. ostianus*, *A. persii*, *A. sclerotiorum*, *A. sesamicola*, and *A. westlandensis* contain a truncated version of the cluster that lacks many of the genes involved in OTA biosynthesis, which might be related to their inability to produce OTA. The gain/loss pattern is different in all species, which suggests that the genetic evolution of this region might be due to independent events.

## 1. Introduction

Ochratoxin A (OTA) is one of the most important mycotoxins due to its ubiquity as a natural contaminant in food and feed, and it exerts several toxic effects that pose clear risks to human and animal health [1]. Traditionally, cereals [2], grapes and their derivatives [3], and coffee [4] have been considered the main sources of OTA in the human diet. However, new insights into OTA occurrence have revealed that dietary exposure to this toxin might also be of importance in other products, such as preserved meat and cheese [5].

The evidence of OTA production was first reported in a culture of *Aspergillus ochraceus* (section *Circumdati*) [6], and for a long time, this species was considered the main OTA producer worldwide. Since then, many other *Aspergillus* and *Penicillium* species have been reported to be capable of synthesizing OTA, and currently, the list of OTA-producing species is rapidly increasing. In an extensive morphological, metabolic, and phylogenetic study, Frisvad et al. [7] described new species included in *Aspergillus* section *Circumdati*, and some of them were not included in the *A. ochraceus* clade. Several studies have indicated that two of the new species, *A. westerdijkiae* and *A. steynii*, are by far the most important OTA producers in this section, and several isolates previously identified as *A. ochraceus*, including the type species and the original OTA-producing strain, were reidentified as *A. westerdijkiae* [7,8,9]. Since then, different studies have extended the knowledge regarding the taxonomy of this section, and currently, 27 species are accepted in *Aspergillus* section *Circumdati* [10]. Recently, Houbraken et al. [11] included new taxonomical groups in the genus *Aspergillus* and separated section *Circumdati* into three series: *Circumdati*, *Sclerotiorum*, and *Steyniorum*. However, there is considerable controversy about the potential of all *Aspergillus* section *Circumdati* species to produce OTA. This ability has been revised in several studies, but it seems to be inconsistent in many species, and no clear conclusions have been reached except for the abovementioned cases (*A. steynii* and *A. westerdijkiae*) [10,12]. *Aspergillus* section *Nigri* also contains important OTA-producing species, such as *A. carbonarius*, *A. niger*, and *A. welwitschiae* [13], whereas, in the *Penicillium* genus, the most important OTA producers are *P. nordicum* and *P. verrucosum* [14]. The species *Aspergillus alliaceus*, included in section *Flavi* is also able to synthetize this toxin [15].

Despite the importance of OTA in food safety, its biosynthetic pathway is not entirely clear and some enzymatic steps are not fully understood [16]. This fact has complicated studies on the molecular and genetic basis of OTA production. For a long time, only a few isolated genes were demonstrated to be involved in OTA biosynthesis in *Aspergillus* species, as well as two small clustered regions in *Penicillium* [17]. However, in the last few years, the rapid evolution of molecular biology techniques, including next-generation sequencing, has significantly increased the number of available genome sequences of OTA-producing species, most of which have been deposited in databases. Therefore, in the last decade, many studies have focused on unraveling the genetic basis of OTA production. A clustered region of five genes is now known to be involved in OTA biosynthesis, and the same conserved synteny is maintained in both *Aspergillus* species (*A. westerdijkiae*, *A. steynii*, *A. niger*, *A. welwitschiae*, and *A. carbonarius*) and *Penicillium nordicum* [18,19]. These five genes encode different enzymes, including a halogenase (HAL), a cytochrome P450 monooxygenase (p450), a non-ribosomal peptide synthetase (NRPS), and a polyketide synthase (PKS) that can catalyze most of the steps of the OTA biosynthetic pathway, as well as a bZIP transcription factor (bZIP) that can regulate the expression of the rest of the clustered genes [20,21]. However, more studies are necessary to resolve existing contradictions on some processes of the biosynthetic pathway, to identify genes that might also be involved in OTA biosynthesis, and to explore how the route is fully regulated [20,21].

As mentioned before, the significance of the *Aspergillus* section *Circumdati* species as OTA producers has not been clearly established. Thus, the aim of this work was to perform an in-depth analysis of the molecular basis of OTA production in the most relevant *Aspergillus* section *Circumdati* species. The sequences of the complete biosynthetic clusters of *A. westerdijkiae* and *A. steynii* were obtained by our group in a previously published paper [18]. In this work, these regions were compared to those present in some relevant *Aspergillus* section *Circumdati* species. The genomes of *A. affinis, A. cretensis*, *A. muricatus*, *A. ostianus*, *A. persii*, *A. roseoglobulosus*, *A. sclerotiorum*, *A. sesamicola*, *A. subramanianii*, and *A. westlandensis* were retrieved from the JGI database, whereas *A. elegans* and *A. melleus* complete genomes were obtained in this work. The putative OTA biosynthetic cluster region of five *A. ochraceus* strains was also sequenced.

## 2. Results

### 2.1. Analysis of the OTA Biosynthetic Cluster in *Aspergillus* Section Circumdati Species Containing the Complete Cluster

The *Aspergillus elegans* genome was completely sequenced in this work and deposited in the NCBI database (BioProject number PRJNA679179). The total genome size was 36.78 Mb, and it was assembled into 8 contigs with a mean length of 4,597,187 bp. The BLAST search performed revealed a long region of 23680 bp that shared very high identity (99%) with the complete OTA biosynthetic cluster of *A. steynii*. When both sequences were aligned, only 120 bp differed between them, and most of these changes occurred in intergenic regions or gene introns. The presence of the complete biosynthetic OTA cluster was also demonstrated in other *Aspergillus* section *Circumdati* species whose genomes, including *A. affinis*, *A. cretensis*, *A. muricatus*, *A. pulvericola*, *A. roseoglobulosus*, and *A. subramanianii*, are available in the JGI database. Table 1 includes the most important features of these regions. The sequences of these clusters were compared with the OTA biosynthetic clusters described in the other section *Circumdati* species. In general, all of the regions found in this work shared higher identity with the corresponding cluster from *A. steynii* than that from *A. westerdijkiae*. The comparison performed revealed that most of the changes found in DNA sequences occurred in gene introns or intergenic sequences that, theoretically, do not affect gene expression. The bZIP binding sites required to activate gene transcription were also found upstream of the four genes in all of the studied species. The sequences of the HAL, BZIP, P450, NRPS, and PKS proteins were predicted from all of the genes of the biosynthetic cluster in all seven species. The changes in DNA sequences were neither modifications in the reading frames nor internal stop codons, and no important changes in the predicted proteins were found that might have affected any of the essential motifs. The sequences of the complete biosynthetic clusters, together with their corresponding gene features, were deposited in the NCBI databases, and their accession numbers are indicated in Table 1. Taking all of these aspects into account, the OTA biosynthetic cluster in *A. affinis*, *A. cretensis*, *A. elegans, A. muricatus, A. pulvericola, A. roseoglobulosus,* and *A. subramanianii* seems to be perfectly functional. Therefore, these species might be capable of producing OTA. Moreover, in all cases, the flanking regions corresponded to genes that encoded an alpha/beta hydrolase and an oxidoreductase, and therefore, the OTA biosynthetic cluster was in the same location in all species.

### 2.2. Analysis of the OTA Biosynthetic Cluster in *Aspergillus* Section Circumdati Species Containing the Truncated Cluster

**Figure 1:**
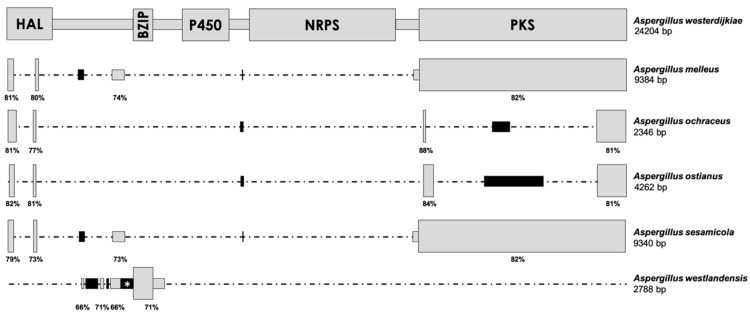
Comparison of the OTA biosynthetic cluster in *A. westerdijkiae* (above) with the corresponding regions found in *A. melleus*, *A. ochraceus*, *A. ostianus*, *A. sesamicola*, and *A. westlandensis*. The schemes are drawn at scale, except in the cases of black regions marked by an asterisk (*), which are slightly longer than represented. The length of the complete region in fungal genomes is indicated below the corresponding species name. Gray regions correspond to sequences that share high identity with the OTA biosynthetic cluster and are located under the corresponding homologous region in *A. westerdijkiae*. Black regions share no identity with any sequence available in databases. The percentages below the boxes indicate the identity of the region with *A. westerdijkiae*. HAL = halogenase-, BZIP = bZIP transcription factor-, P450 = cytochrome P450 monooxygenase-, NRPS = non-ribosomal peptide synthetase-, and PKS = polyketide synthase-encoding genes.

The primer pair designed on the basis of the flanking regions of the OTA biosynthetic cluster of *A. westerdijkiae* and *A. steynii* yielded a clear band using the DNA of all *A. ochraceus* strains tested. The fragment was first sequenced in *A. ochraceus* CECT 2093, which is known to be a nonproducing strain [9]. After four rounds, a sequence of 3330 bp was obtained. This approach was also used to sequence the fragment amplified in *A. ochraceus* CECT 2969, CECT 2970, PDF2, and R1, and a similar length was obtained in all cases, with high identity values with the sequence obtained in *A. ochraceus* CECT 2093 (97–99%). When the sequence of this region was compared to the biosynthetic cluster of *A. westerdijkiae,* high identity values were observed in some regions (78–85%), although many parts of the cluster were absent in the *A. ochraceus* amplified region. A scheme that compares both regions is shown in Figure 1. Only four small parts of the biosynthetic cluster remained in *A. ochraceus* and corresponded to some parts of the HAL- and PKS-encoding genes. Moreover, a sequence gain seems to have occurred, since two additional regions were also found that shared no identity with any sequence available in databases. The same pattern of sequence gain/loss was found in the *A. ostianus* genome, although the length of each fragment was slightly different (Figure 1).

The complete genome of *A. melleus* was sequenced in this work in order to study the putative cluster involved in OTA biosynthesis in this species. The accession number of the BioProject in the NCBI database is PRJNA679179. A genome size of 38.85 Mb was found, and the genome was assembled into 11 contigs (mean length of 3,531,893 bp). After a BLAST search in the NCBI database, a region of 9384 bp was found to show identity with the OTA biosynthetic cluster in *A. westerdijkiae*. A comparison of both regions is shown in Figure 1. In this case, the region also appears to have lost some parts of the biosynthetic cluster, although the deletion pattern is different from that observed in *A. ochraceus*. In that case, the complete CDS of the PKS-encoding gene remains in the genome, together with some small parts of the HAL-encoding gene and a short sequence of the intergenic spacer between this gene and the bZIP transcription factor-encoding one. Three unknown small sequences also appear in this region in the *A. melleus* genome. This gain/loss pattern was also observed in the putative OTA cluster region found in the *A. sesamicola* genome (Figure 1).

The remaining parts of the OTA biosynthetic cluster found in *A. westlandensis* widely differ from those reported in the other species, and it is the only one that has not maintained the 3′-end of the PKS-encoding gene. In this case, the complete BZIP-encoding gene is present, as well as other intergenic regions that are located close to this gene. The gain of some regions was also observed (Figure 1).

**Figure 2:**
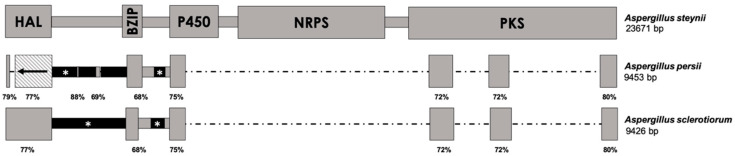
Comparison of the OTA biosynthetic cluster in *A. steynii* (above) with the corresponding regions found in *A. persii* and *A. sclerotiorum*. The schemes are drawn at scale, except in the cases of black regions marked by an asterisk (*), which are slightly longer than represented. The length of the complete region in fungal genomes is indicated below the corresponding species name. Gray regions correspond to sequences that share high identity with the OTA biosynthetic cluster and are located under the corresponding homologous region in *A. steynii*. Black regions show no identity with any sequence available in databases. The striped square represents an inverted region. The percentages below the boxes indicate the region’s identity with *A. steynii*. HAL = halogenase-, BZIP = bZIP transcription factor-, P450 = cytochrome P450 monooxygenase-, NRPS = non-ribosomal peptide synthetase-, and PKS = polyketide synthase-encoding genes.

All of the aforementioned species that contain a truncated version of the OTA biosynthetic cluster are classified in series *Circumdati* and share higher identity with the corresponding region of the *A. westerdijkiae* genome. However, the rest of the truncated versions of the OTA cluster found in *Aspergillus* section *Circumdati* species show the highest identity with the genomic region of *A. steynii*, and therefore, comparisons of this sequence were performed. This second group of species that contains the truncated cluster belongs to series *Sclerotiorum*. The presence of the OTA biosynthetic cluster within the genome of *A. sclerotiorum* was also studied. The *A. sclerotiorum* genome was found to contain a region of 9426 bp with different sequences that are similar to the OTA biosynthetic cluster of *A. steynii*, with identity values that range between 68 and 82% (Figure 2). These regions correspond to the complete CDS of the HAL-encoding gene, parts of the genes encoding BZIP and P450, and three short sequences of the PKS-encoding gene, including its 3′-end. The intergenic regions between HAL and bZIP as well as between BZIP and P450 are slightly longer, and no identity at all was found between them and those present in the *A. steynii* OTA cluster.

The partial OTA cluster found in *A. persii* maintained the same regions of the BZIP-, P450-, and PKS-encoding genes as *A. sclerotiorum*, although a phenomenon of genetic inversion was observed in the HAL-encoding gene (Figure 2). Whereas the 3′-end of the CDS was conserved in the correct direction, the 5′-end was found to be inverted from its original form, indicating that, apart from gain/loss events, the OTA cluster of *A. persii* underwent a rearrangement event.

The flanking regions of the partial OTA cluster were studied in the genomes of the species analyzed in this work that contained either the complete or the truncated version. In general, both genes encoding an alpha/beta hydrolase and an oxidoreductase were maintained, indicating that the location of the cluster is the same in all of the species. The only exception was found in the *A. sclerotiorum* genome, which maintained the alpha/beta hydrolase-encoding gene in one of the flanks, but it also lost the gene corresponding to the oxidoreductase-encoding gene.

### 2.3. Phylogenetic Analysis of Aspergillus Section Circumdati Related to the Presence of the OTA Biosynthetic Cluster

**Figure 3:**
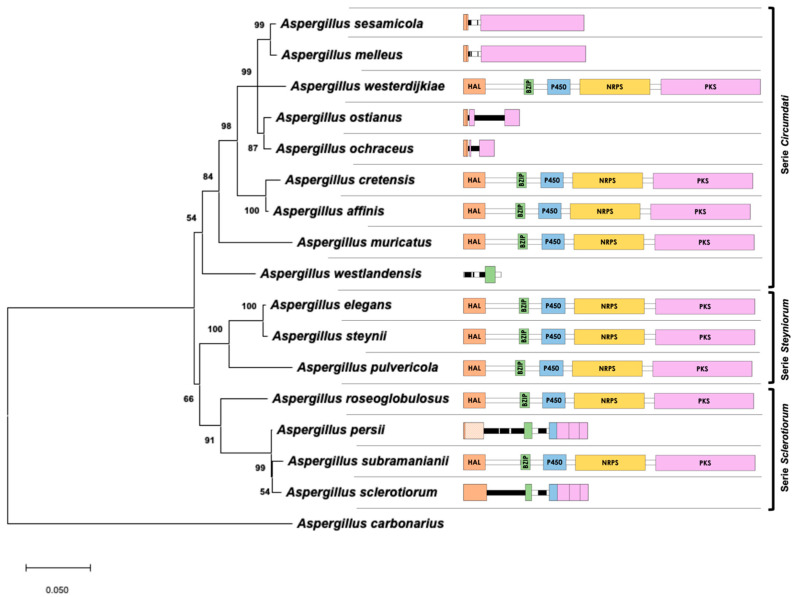
Maximum likelihood tree derived from a combination of β-tubulin, ITS1-5.8S-ITS2, and calmodulin data. The tree with the highest log-likelihood (−5103.86) is shown. Branch lengths are measured in the number of base substitutions per site. Bootstrap values are shown next to the branches. A scheme (drawn at scale) of the putative region that includes the biosynthetic cluster in the genome of each taxon is located close to the corresponding branch.

The evolutionary history, which was inferred using a maximum likelihood (ML) approach with the combined dataset of β-tubulin/ITS1-5.8S-ITS2/calmodulin, is shown in Figure 3. The best model to describe the substitution pattern of the sequences was Tamura Nei, and the rate variation among sites was modeled with a gamma distribution (shape parameter = 0.31). This phylogeny, based on the ML method, is a reflection of the real phylogeny of *Aspergillus* section *Circumdati* that was previously reported by other authors [7,22], and the branches are strongly supported by high bootstrap values. Near the branches, Figure 3 also shows a schematic that represents the sequences of the biosynthetic cluster present in the genome of the corresponding species. It is clear that the presence/absence of the complete cluster does not completely reflect the phylogeny of the section. Therefore, the gain/loss of the genes involved in OTA biosynthesis might be due to independent events.

Whereas the complete OTA biosynthetic region was maintained in all analyzed species of series *Steyniorum,* the presence of a partial cluster was frequent in series *Circumdati* and *Sclerotiorum*. Moreover, similar gain/loss patterns were observed in closely related species, such as *A. sesamicola* and *A. melleus*, *A. ochraceus* and *A. ostianus*, and *A. persii* and *A. sclerotiorum*.

**Figure 4:**
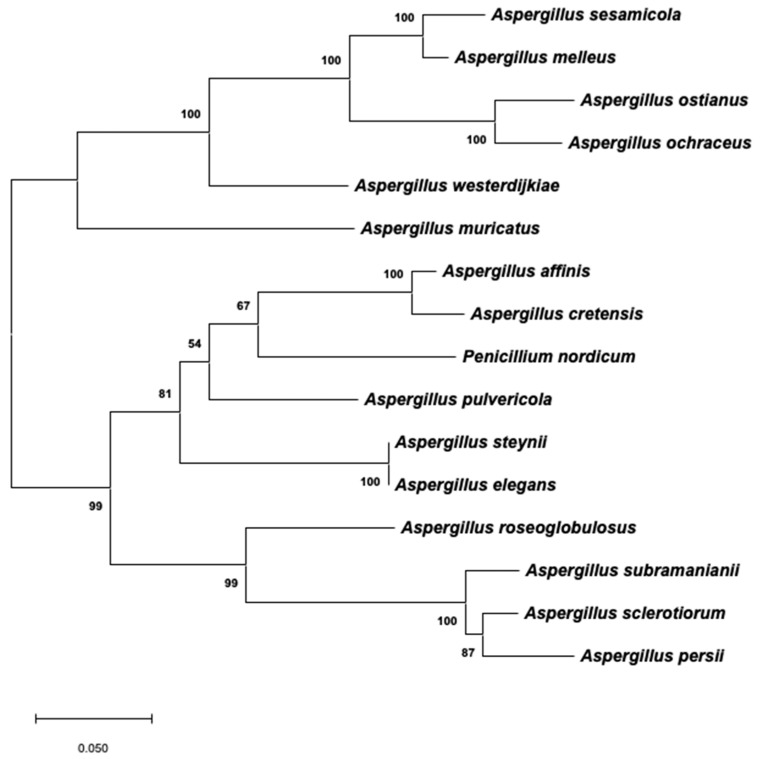
Maximum likelihood tree derived from the 3′-end of the PKS-encoding gene sequences. The tree with the highest log likelihood (−3842.76) is shown. Branch lengths are measured in the number of base substitutions per site. Bootstrap values are shown next to the branches.

A similar phylogeny using the same species was constructed on the basis of the partial 3′-end of the PKS-encoding gene that was found to remain in all of the species evaluated, except in the case of *A. westlandensis*. The corresponding ML tree is shown in Figure 4. Tamura 3-parameter was the best evolutionary model to describe the substitution pattern, and the rate variation among sites also followed a gamma distribution with a shape parameter of 0.61. As mentioned in the Materials and Methods section, it was not possible to include an appropriate outgroup taxon. Therefore, this unrooted phylogeny is not strictly comparable to that constructed using the β-tubulin/ITS1-5.8S-ITS2/calmodulin dataset. However, some conclusions can be made independently for the two main branches. It is clear that the topology of this tree reveals a higher evolution rate in the species that contain a nonfunctional version of the OTA cluster. In series *Circumdati*, the last branches corresponded to *A. sesamicola*, *A. melleus*, *A. ostianus*, and *A. ochraceus*, and the same situation arose in series *Sclerotiorum*, in which *A. persii* and *A. sclerotiorum* clustered together in the last branch. All six of the mentioned species contain a truncated version of the OTA cluster. It is important to highlight the cluster formed by *A. affinis* and *A. cretensis*, which were classified as series *Circumdati* but grouped together with the members of series *Steyniorum* in this tree. Moreover, the OTA-producing species *P. nordicum* was also included in this cluster.

**Figure 5:**
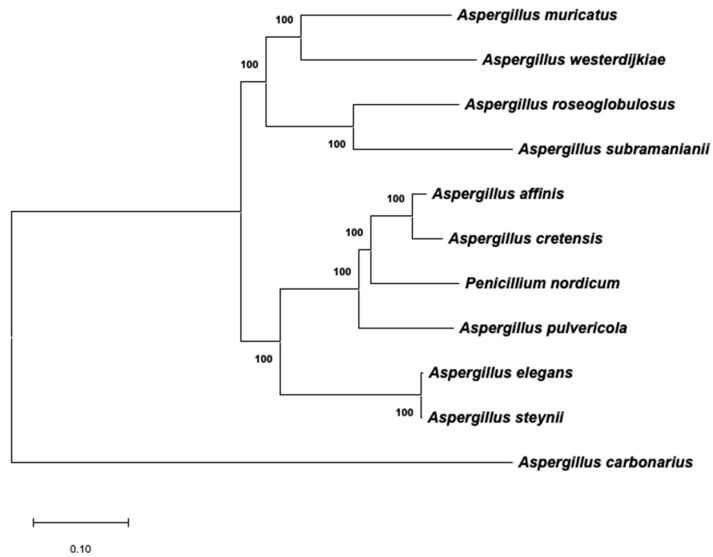
Maximum likelihood tree derived from the sequences of the complete OTA biosynthetic cluster. The tree with the highest log-likelihood (−5103.86) is shown. Branch lengths are measured in the number of base substitutions per site. Bootstrap values are shown next to the branches.

The complete sequence of the OTA biosynthetic cluster found in *Aspergillus* section *Circumdati* species was also analyzed using an ML phylogeny. The corresponding sequence of *P. nordicum* was also included in the analysis. The ML tree obtained is shown in Figure 5. The General Time-Reversible model had the best ability to describe the substitution pattern. A discrete gamma distribution was used to model the evolutionary rate differences among sites (alpha parameter = 2.01) and allowed for some sites to be evolutionarily invariable (27.66%). This phylogenetic analysis revealed that the OTA clusters in *A. westerdijkiae* and *A. muricatus* were more related to that of series *Sclerotiorum*, since they diverged together from the same cluster. However, as occurred in the case of the analysis performed with the 3′-end of the PKS-encoding gene, *A. affinis* and *A. cretensis* were closer to the members of series *Steyniorum* than to their closely related species *A. westerdijkiae* and *A. muricatus*. Moreover, the two species clustered together with *A. pulvericola* and *P. nordicum* in a branch supported by high bootstrap values. This fact supports the hypothesis that *P. nordicum* acquired the biosynthetic cluster via horizontal transfer from an *Aspergillus* species included in section *Circumdati* [17] and reduces the number of the most probable donors to these three species.

## 3. Discussion

*Aspergillus* section *Circumdati* includes 27 species, which are able to produce a large variety of secondary metabolites [10]. As mentioned above, there is a lot of controversy regarding OTA production by *Aspergillus* section *Circumdati* species. In this work, the study of fungal genomes of different species revealed interesting data about the presence of the complete or truncated OTA biosynthetic cluster that might be related to their ability/inability to synthesize the toxin.

The inability to produce mycotoxins due to the deletion of some genes of the biosynthetic cluster has been frequently reported in the *Aspergillus* genus by several authors. Some strains of *A. oryzae* are not able to produce aflatoxins, and the loss of several genes involved in their synthesis has been reported [23]. Similarly, Adhikari et al. [24] reported that a number of *A. flavus* strains were not able to produce aflatoxins due to deletions of some genes involved in early biosynthesis stages. A similar situation occurred in the case of the fumonisin B2 biosynthetic cluster in *Aspergillus niger* aggregate species [19,25]. Therefore, deletion of parts of the biosynthetic cluster seems to be a common path related to the loss of mycotoxigenic ability in these species.

The partial deletion of the cluster of genes involved in OTA biosynthesis has also been recently studied in *Aspergillus niger* aggregate species [19,25]. In all cases, the presence of this truncated cluster is related to the inability of the isolates to produce the toxin, and, to our knowledge, it seems to occur in most *A. niger* and *A. welwitschiae* strains and all *Aspergillus tubingensis*. In all cases, this long deletion in *Aspergillus niger* aggregate species is accompanied by the maintenance of a region that is approximately 600 bp long, at the end of the biosynthetic cluster, which corresponds to the 3′-region of the PKS gene. This aspect might be assumed to be related to a single deletion event that eliminated almost the complete cluster. However, in the case of the species of *Aspergillus* section *Circumdati,* some parts of the genes or intergenic regions of the biosynthetic cluster remain in the genome, and these parts are different in each species, which could be related to subsequent gains and losses over time. These multiple independent loss events have also been described in the deletion of the bikaverin biosynthetic cluster in *Botrytis* spp. [26].

*Aspergillus ochraceus* was the first reported OTA-producing species and, for a long time, it was considered an important contributor to OTA levels in foodstuffs. However, due to frequent changes in taxonomy, its relative importance drastically dropped with the emergence of *A. westerdijkiae* and *A. steynii* as the main producers in section *Circumdati* [7,9]. In this work, *A. ochraceus* was analyzed to elucidate the genes involved in OTA production in this species. However, surprisingly, the typical clusters that are present in both *Aspergillus* and *Penicillium* species, as reported previously by our group [18], were not found, and only small remaining parts of some genes were present in the *A. ochraceus* genome. Due to the importance of this species as an OTA producer, these results were confirmed in five different strains from different origins. The same deletion patterns were found in all of the isolates studied, indicating a consistent lack of the ability to produce OTA in this species. All of these isolates had been previously classified as nonproducing strains of *Aspergillus ochraceus* [9]. However, some recently published papers have described some *A. ochraceus* isolates that are able to produce OTA, although it is true that they do so inconsistently and yield very low levels. As mentioned before, identification of *Aspergillus* section *Circumdati* species is especially difficult and often requires the application of molecular techniques [8,12]. Some papers that have recently described these OTA-producing isolates have performed their identification based only on morphological characteristics, which has been demonstrated to be insufficient [27,28,29,30,31,32,33]. Therefore, it might be possible that they were misidentified and were not really *A. ochraceus*, but instead other species, such as *A. steynii* or *A. westerdijkiae*, that are more relevant OTA producers in foodstuffs. Similarly, Wang et al. [34] recently published the genome of *A. ochraceus* fc-1 and reported the presence of the complete OTA biosynthetic cluster and the ability of this strain to produce the toxin. However, the analysis of this genome, which is available on the NCBI database, revealed a misidentification of the strain fc-1 that should be reclassified as *A. westerdijkiae* on the basis of β-tubulin and ITS1-5.8S-ITS2 sequences. On the other hand, other papers that recently described *A. ochraceus* as an OTA producer evaluated toxin concentrations using HPLC methods and have detected extremely low levels of OTA in the medium, which have been very close to detection limits [35]. It is known that some fungi are able to form some compounds that might be misidentified as OTA using HPLC. This was the case for *A. tubingensis*, which led to the species being considered an OTA producer for a long time [25,36].

Apart from *A. ochraceus*, six other *Aspergillus* section *Circumdati* species contain a truncated version of the OTA biosynthetic cluster: *A. melleus*, *A. ostianus*, *A. persii*, *A. sclerotiorum*, *A. sesamicola*, and *A. westlandensis*. The ability of these species to produce OTA has been frequently described as inconsistent, and previous studies have indicated that only trace levels of the toxin could be detected, even in highly permissive conditions [7,10,12]. Our results demonstrate that these species may not be OTA producers since they do not contain the biosynthetic cluster in their genomes.

Several hypotheses on the reason why mycotoxin biosynthetic clusters are lost over evolution have been proposed, and their common tenet is that adaptation to new situations might be decisive. It is well known that gene loss has been crucial to fungal evolution and, in some cases, is correlated with shifts in ecological niches or association with a host [37]. Campbell et al. [26] suggested that the acquisition of the bikaverin cluster might have provided a selective advantage that allowed *Botrytis* spp. to survive as saprotrophs in the soil. However, as *Botrytis* genomes evolved toward virulent, endophytic strains, bikaverin production was no longer retained. A similar fact was observed in the case of *Penicillium roqueforti* and its inability to produce mycophenolic acid due to a short deletion of 174 bp in the biosynthetic cluster [38]. All of the strains that do not harbor the capacity to produce this toxin are isolated from blue cheese. The authors hypothesized that these strains may no longer require this trait, which has a high energetic cost, as an evolutionary advantage to compete with other organisms. For this reason, they do not maintain the ability to produce mycophenolic acid. This idea of ecological adaptation due to the domestication of strains was also proposed by Martín and Liras [39] regarding the inability of *P. roqueforti* to produce meleagrin and neoxalin. These authors affirmed that the production of these toxins did not confer an ecological advantage to the fungus, although they asserted that the deletion of the genes is not a recent event, since the deletion is consistent in isolates from different origins and types of cheese.

In the last few years, different studies have been published regarding the relative significance of *Aspergillus* spp. in human pathologies. In this context, some *Aspergillus* section *Circumdati* species, including *A. melleus*, *A. ochraceus*, *A. persii*, and *A. sclerotiorum*, were described as causal agents of onychomycosis [40]. All of these species contain only a short fragment of the OTA cluster in their genomes, and adaptation to this unusual habitat might be related to the loss of their ability to produce the toxin because it does not have a useful function. Because they were recently described as new species in *Aspergillus* section *Circumdati*, there are no reports on the occurrence of *A. westlandensis* or *A. sesamicola*, and no hypothesis addresses their inability to produce OTA. However, it is important to highlight *A. westlandensis*, since it presents a unique pattern in the remaining regions of the OTA cluster that might be related to independent events during its speciation.

The analysis of fungal genomes revealed the presence of the complete OTA biosynthetic cluster in seven *Aspergillus* section *Circumdati* species, which, together with *A. steynii* and *A. westerdijkiae*, should be considered as possible OTA producers within this section. As mentioned above, several studies have reported the ability of these species to produce OTA. In the most recently published study, Visagie et al. [10] indicated that *A. affinis*, *A. cretensis*, *A. muricatus*, *A. pulvericola*, and *A. roseoglobulosus* are consistent OTA producers, since the authors detected large amounts of OTA in fungal cultures. This fact is in agreement with the results of this work, in which the presence of a putative functional OTA cluster was detected in their genomes, which indicates their potential ability to synthesize the toxin. To date, few reports have studied the natural occurrence of these species, which, in some cases, is due to their recent description. For example, *A. pulvericola* has only been reported in old, deteriorated manuscripts [41], whereas *A. affinis* has been detected in decomposing leaves and in a cave environment [42,43]. Therefore, it is essential to perform exhaustive surveys to study the presence of these potential OTA-producing species in foodstuffs to establish the risk that they might pose to food security and human health.

*Aspergillus subramanianii* and *A. elegans* are considered inconsistent OTA producers because the toxin has been detected in only some cases and at low levels in fungal cultures [9,10]. The in silico analysis performed in this work suggests that the enzymes encoded by the clustered genes that are involved in toxin production might be functional. However, some atoxigenic strains have also been described in *A. carbonarius*, which contains no large deletions in the OTA biosynthetic cluster. However, all of the genes are downregulated, even under highly permissive conditions [44]. Even containing the complete cluster in their genomes, some atoxigenic strains of *A. westerdijkiae* and *A. steynii* have also been described, and no expression has been detected in the five genes involved in OTA biosynthesis [9,18]. Furthermore, some authors have suggested that post-transcriptional modifications might also interfere in the regulation of mycotoxin biosynthesis. These mechanisms include microRNA and virus-mediated modulation, among others [45,46]. Thus, the ability of *A. subramanianii* and *A. elegans* to produce OTA should be studied in detail to unravel whether some regulatory mechanisms are affected.

As mentioned before, Adhikari et al. [24] related the inability to produce aflatoxins to deletions of some genes in the biosynthetic cluster. The deletion patterns allowed *A. flavus* isolates to be classified into six different groups that revealed a consistent evolutionary relationship between most of the genotypes, suggesting distinct deletion events for each group. However, in our study, no evident evolutionary relationships were found between *Aspergillus* section *Circumdati* species and the loss of the OTA cluster. In comparing the presence/absence of the biosynthetic cluster in *Aspergillus* section *Circumdati* species with the real phylogeny constructed based on β-tubulin, ITS1-5.8S-ITS2, and calmodulin regions, we observed no obvious patterns, which suggests that the evolution of this region might be due to independent gain/loss events during speciation. However, it is interesting to highlight that all tested species of series *Steyniorum* have maintained the complete OTA biosynthetic cluster, and therefore, it seems to be a common threat in this series. In this work, only one isolate was tested in most of the species. Therefore, it would be interesting to confirm whether deletion patters present intraspecific variability. Another relevant hypothesis that could be made regarding phylogenetic analysis is the origin of the *P. nordicum* cluster, which is known to have acquired the cluster by horizontal transfer from *Aspergillus* species [18]. To date, the highest identity found is between *P. nordicum* and *A. steynii* OTA clusters, and the hypothesis indicates that this section *Circumdati* species might have been the donor to the *Penicillium* species. However, phylogenetic analysis clustered *P. nordicum* with *A. affinis* and *A. cretensis*, showing that they share extremely high identity in their OTA biosynthetic regions (83% and 80%, respectively). Therefore, these results suggest that the transference of the OTA biosynthetic cluster to the *Penicillium* genus might have occurred from one of these two *Aspergillus* section *Circumdati* species.

The study of the gain/loss pattern of the OTA biosynthetic cluster revealed that, except for *A. westlandensis*, a short region of the 3′-end of the cluster was maintained in all species of *Aspergillus* section *Circumdati* and in section *Nigri*. The phylogenetic analysis performed using the sequences of this region in *Aspergillus* section *Circumdati* reflects that the evolutionary history of the section, as revealed by the analysis of β-tubulin/ITS1-5.8S-ITS2, is similar in some groups but widely different in others. This aspect might suggest that the cluster was present in the genome of the common ancestor of *Aspergillus* section *Circumdati* species and then evolved independently during speciation. The reason that both nonproducing *Aspergillus* section *Nigri* and *Circumdati* species have maintained the 3′-end of the PKS-encoding gene remains unknown, and its importance needs to be addressed.

As mentioned before, many taxonomical revisions of *Aspergillus* section *Circumdati* have been published in the last few years in which new species were described and some isolates were reclassified. Therefore, currently, it is difficult to accurately determine the habitat of each species. However, many authors have suggested that *A. westerdijkiae* and *A. steynii* are probably the most frequent species that occur in foodstuffs in the section [12,47], whereas the rest of the species occur in other ecological environments [48]. It is not completely clear why fungi produce mycotoxins, but some evidence indicates that they provide a competitive advantage to defend their niche from other species [49]. It might be thought that *A. steynii* and *A. westerdijkiae* have retained their ability to produce OTA to effectively colonize food products, whereas the species more adapted to other less-specialized environments might have lost the cluster to avoid extreme energy expenses due to OTA production.

## 4. Conclusions

The results presented here provide insight into the genetic basis of OTA production in the most important *Aspergillus* section *Circumdati* species. The *Aspergillus ochraceus* strains analyzed in this work did not contain the OTA biosynthetic cluster in their genome. Therefore, this species might be excluded as an OTA producer, in which case studies on the occurrence of this fungus are no longer relevant. Moreover, it is crucial to carry out large surveys regarding the occurrence of species that are demonstrated to be potential OTA producers in food products to unravel their relative importance in food safety, such as *A. affinis*, *A. cretensis*, *A. elegans*, *A. muricatus*, *A. pulvericola*, and *A. subramanianii*. It would be also interesting to confirm the lack of intraspecific variation and whether all the isolates of these species are potential OTA producers. Due to the fact that the loss of the cluster seems to be the most frequent mechanism of the inability to produce OTA in *Aspergillus* species, this region might be used to design molecular detection methods based on PCR for the early detection of OTA producers in foodstuffs.

## 5. Materials and Methods

### 5.1. Putative OTA Cluster Determination in Aspergillus ochraceus Strains

Five *Aspergillus ochraceus* strains were used to study the presence of the OTA biosynthetic cluster in fungal genomes. These strains included the type strain CECT 2093, other isolates obtained from Spanish Type Culture Collection (CECT 2969 and CECT 2970), and two *A. ochraceus* obtained from previous works in our laboratory (PDF2 and R1). The inability of these strains to produce has been demonstrated before [9].

Genomic DNA was isolated from 3-day-old cultures in Sabouraud Broth. Mycelia were harvested by filtration using Whatman Paper Nº1, frozen with liquid nitrogen, and ground using a mortar and a pestle. DNA isolation was performed using the protocol described elsewhere [50]. DNA concentrations were determined using a NanoDrop^®^ ND-1000 spectrophotometer (Nanodrop Technologies, Wilmington, NC, USA).

A new set of degenerate primers were designed on the basis of the sequences of flanking genes of the OTA biosynthetic cluster in *A. steynii* and *A. westerdijkiae*, and they amplified the region located between the 3′-end of the alpha/beta hydrolase and the 5′-end of the oxidoreductase. Therefore, the primer pair interHID-F (5′-CGTGTGATTGGGAKCGG-3′) and interOXI-R (5′-CTGGSGATGGRAGTCGA-3′) was used to amplify the region in *A. ochraceus* strains using the following PCR conditions: 1 cycle of 5 min at 95 °C; 30 cycles of 1 min at 94 °C, 1 min at 63 °C, and 2 min at 72 °C; and a final extension of 5 min at 72 °C. The amplification reactions were carried out in volumes of 25 μL, containing 100 ng of sample DNA, 1 μL of each primer (20 μM; Metabion, Planegg, Germany), and 12.5 μL of NZYTaq II 2x Green Master Mix (Nzytech, Lisbon, Portugal).

PCR products were detected in 1.5% agarose gels in TAE 1X buffer (40 mM Tris-acetate and 1.0 mM EDTA) with 0.002% GreenSafe Premium stain (Nzytech, Lisbon, Portugal). The NZYDNA Ladder VII (Nzytech, Lisbon, Portugal) was used as a molecular size marker. PCR products were purified using the kit NZYGelpure (Nzytech, Lisbon, Portugal).

Due to the length of the PCR products obtained and the impossibility of obtaining a sequence of quality using the primer interOXI-R, different rounds of sequencing had to be performed. The first sequencing step was carried out using the primer interHID-F, and the complete sequence was subsequently obtained using interOCRAseq1 (5′-GACACCGAGTCATGCAGCGTC-3′), interOCRAseq2 (5’-GGCTGTTGTTTATCTGATCTGACA-3′), and interOCRAseq3 (5′-GACTCGTTAGTGGCAGTGGACTT-3′). PCR products were sequenced in Macrogen Facilities (Madrid, Spain) using an ABI 3730XL DNA sequencer (Applied Biosystems, Foster City, CA, USA). Sequences were edited with Unipro UGENE bioinformatics software version 1.25.0 (Unipro, Novosibirsk, Russia) [47].

### 5.2. Genome Sequencing of Aspergillus melleus and Aspergillus elegans

The complete genome sequence of *A. elegans* IMI 345568 and *A. melleus* CBS 546.65 were obtained by next-generation sequencing. Genomic DNA was isolated as described in the previous subsection. A hybrid sequencing approach was applied to obtain the highest quality results. This method combined the PacBio long-read platform and Illumina sequencing. The DNA sample was used for library construction using the PacBio SMRTbell Library Preparation Kit (Pacific Biosciences, Menlo Park, CA, USA) with a 20 kb insert size and was subsequently analyzed in a PacBio RSII sequencer (Pacific Biosciences, Menlo Park, CA, USA). In the case of Illumina data, the sequencing library was prepared using the TruSeq DNA Whole genome library preparation kit (Applied Biosystems, Foster City, CA, USA), and sequencing was performed in a NovaSeq 6000 system (Applied Biosystems, Foster City, CA, USA). The de novo assembly of PacBio reads was performed using the Hierarchical Genome Assembly Process (HGAP4). Then, Illumina reads were applied for accurate genome sequence using Pilon.

### 5.3. Sequence Analysis and Putative Cluster Identification in Other Aspergillus Section Circumdati Species

The complete genome sequences of Aspergillus affininis CBS 129190, Aspergillus cretensis CBS 112802, Aspergillus muricatus CBS 112808, Aspergillus ostianus CBS 103.07, Aspergillus persii CBS 112795, Aspergillus pulvericola CBS 137327, Aspergillus roseoglobulosus CBS 112800, A. sclerotiorum CBS 549.65, Aspergillus sesamicola CBS 137324, Aspergillus subramanianii CBS 138230, and Aspergillus westlandensis CBS 123905 are available on the JGI Genome Portal website and are a part of the Aspergillus whole-genome sequencing project started in 2013 by Scott E. Baker. The correct links to access these genomes are included in Table S1. The presence of the putative region containing the OTA cluster in these fungal genomes was tested using the JGI Alignment Search BLAST tool, available on the website, using the sequence of the OTA biosynthetic cluster of A. steynii CBS 112.812 (accession number MG701897) as the query.

### 5.4. Bioinformatics Analysis for Ochratoxin A Biosynthetic Cluster Comparisons

The putative regions corresponding to the biosynthetic cluster in *A. steynii* and *A. westerdijkiae* and the truncated cluster obtained in *A. melleus*, *A. ochraceus*, *A. ostianus*, *A. persii*, *A. sclerotiorum*, and *A. sesamicola* were compared using the bl2seq tool available at the NCBI website. The percentage of identity of the different regions of the sequences was also obtained using this tool.

Other bioinformatics analyses were performed if the complete cluster was observed in fungal genomes. Identity calculations and intron locations were carried out using Unipro UGENE 1.25.0 [51]. Protein sequence predictions were carried out using the Translate tool available at the Bioinformatics Resource Portal of the Swiss Institute of Bioinformatics (https://web.expasy.org/translate/), whereas domain analyses were performed with the PFAM 31.0 software available on the EMBL webpage (http://pfam.xfam.org).

### 5.5. Phylogenetic Analysis

DNA sequences were aligned using the iterative MUSCLE algorithm. Subsequently, the relationships among the species were visualized using a maximum likelihood (ML) tree. Three datasets were constructed to study the relationships among the taxa. In all cases, the corresponding sequences of *Aspergillus carbonarius* were used as the outgroup taxon. The first one included a combined dataset with the partial β-tubulin gene, the ITS1-5.8S-ITS2 region, and the partial sequence of the calmodulin gene, because they were reported as phylogenetically informative in evaluating interspecific relationships in *Aspergillus* section *Circumdati.* The sequences used to construct this phylogenetic tree are available in the NCBI database, and their accession numbers are included in Table 2. There were a total of 1233 positions in the final dataset.

The second dataset included a short sequence of the 3′-end of the PKS gene, which was found to be conserved in the genome of all the species analyzed except in *A. westlandensis*. This dataset was constructed with the sequences obtained in this work, as described above, together with others used in previous works in our laboratory [18]. In this case, it was not possible to find an appropriate outgroup taxon because of the high divergence between the sequences in *Aspergillus* section *Circumdati* and *Nigri*, which include other species that are able to produce OTA. Therefore, the tree constructed from the 3′-end of the PKS gene was unrooted. There was a total of 624 positions in this final dataset.

The last dataset corresponds to the sequences of the OTA biosynthetic genes and includes all of the species that were found to contain the complete cluster in their genomes. The corresponding sequence of *A. carbonarius* was used as the outgroup taxon. This final dataset was formed by 19,747 positions.

The data were first evaluated in order to establish the best evolutionary model. Subsequently, an ML tree was constructed using MEGA X [52]. The trees were drawn at scale, with branch lengths measured in the number of substitutions per site. Initial trees for the heuristic search were obtained automatically by Neighbor-Join and BioNJ algorithms applied to a matrix of pairwise distances estimated using the corresponding model. Then, the topology with the best log-likelihood value was selected. All positions containing gaps and missing data were eliminated from the analysis. Bootstrap values were calculated from 1000 replications of the bootstrap analysis to determine the support for the clades.

The analyses involved 17, 16, and 11 nucleotide sequences in the cases of β-tubulin/ITS1-5.8S-ITS2/calmodulin, the 3′-end of the PKS, and the complete cluster datasets, respectively.

## Figures and Tables

**Table 1:** Characteristics of the ochratoxin A (OTA) biosynthetic clusters described in this work. The length of the complete cluster, from the beginning of the halogenase (HAL)-encoding gene to the end of the polyketide synthase (PKS)-encoding gene, is indicated, together with the accession number of the sequence in the NCBI database. The identity of the complete region compared to the reference clusters in *A. steynii* and *A. westerdijkiae* is also indicated.

Species	Cluster Length	Accession Number	Identity with OTA Cluster	
			*A. steynii*	*A. westerdijkiae*
*A. affinis*	23,387	MT955635	75%	67%
*A. cretensis*	23,586	MT955636	73%	66%
*A. elegans*	23,680	MT904985	99%	67%
*A. muricatus*	23,696	MT955638	70%	70%
*A. pulvericola*	23,409	MT904986	74%	66%
*A. roseoglobulosus*	23,599	MT955637	70%	67%
*A. subramanianii*	23,730	MT955639	68%	65%

**Table 2:** Accession numbers in the NCBI database of the sequences used to generate the combined dataset β-tubulin/ITS1-5.8S-ITS2/calmodulin for maximum likelihood phylogenetic analysis.

Species	β-Tubulin	ITS1-5.8S-ITS2	Calmodulin
*Aspergillus affininis* CBS 129190	GU721092	JN882309	GU721091
*Aspergillus cretensis* CBS 112802	AY819977	FJ491572	FJ491534
*Aspergillus elegans* CBS 102.14	EF661349	EF661414	EF661390
*Aspergillus melleus* CBS 546.65	EF661326	EF661425	EF661391
*Aspergillus muricatus* CBS 112808	EF661356	EF661434	EF661377
*Aspergillus ochraceus* CECT 2093	EF661322	EF661419	EF661381
*Aspergillus ostianus* CBS 103.07	EF661324	EF661421	EF661385
*Aspergillus persii* CBS 112795	AY819988	FJ491580	FJ491559
*Aspergillus pulvericola* CBS 137327	KJ775055	KJ775440	KJ775236
*Aspergillus roseoglobulosus* CBS 112800	AY819984	FJ491583	FJ491555
*Aspergillus sclerotiorum* CBS 549.65	EF661337	EF661400	EF661384
*Aspergillus sesamicola* CBS 137324	KJ775063	KJ775437	KJ775233
*Aspergillus steynii* CBS 112.812	EF661347	EF661416	EF661378
*Aspergillus subramanianii* CBS 138230	EF661339	EF661403	EF661397
*Aspergillus westerdijkiae* CECT 2948	EF661329	EF661427	EF661360
*Aspergillus westlandensis* CBS 123905	KJ775065	KJ775433	KJ775229
*Aspergillus carbonarius* NRRL 4849	EF661100	EF661205	EF661168

## Supplementary Materials

The following are available online at https://www.mdpi.com/2072-6651/12/12/754/s1: Table S1: Genomes used in this study, together with their accession links and their years of publication.
