# Supplementary material for: The Genomic Regions That Contain Ochratoxin A Biosynthetic Genes Widely Differ in Aspergillus Section Circumdati Species
Source: Toxins (Basel). 2020 Nov 29;12(12):754. doi: 10.3390/toxins12120754 (PMC7760312; doi:10.3390/toxins12120754)
Supplement: Supplementary file 1 [file toxins-12-00754-s001.pdf]

Supplementary Materials: The Genomic Regions That Contain Ochratoxin A Biosynthetic Genes Widely Differ in *Aspergillus* Section *Circumdati* Species

Jéssica Gil-Serna, Covadonga Vázquez and Belén Patiño

Table S1. Genomes used in this study, together with their accession links and their years of publication.

Genome	Available at	Year
Aspergillus affininis CBS 129190	https://mycocosm.jgi.doe.gov/ Aspaff1/Aspaff1.home.html	2019
Aspergillus cretensis CBS 112802	https://mycocosm.jgi.doe.gov/ Aspcre1/Aspcre1.home.html	2019
Aspergillus muricatus CBS 112808	https://mycocosm.jgi.doe.gov/ Aspmuri1/Aspmuri1.home.html	2016
Aspergillus ostianus CBS 103.07	https://mycocosm.jgi.doe.gov/ Asposti1/Asposti1.home.html	2014
Aspergillus persii CBS 112795	https://mycocosm.jgi.doe.gov/ Aspper1/Aspper1.home.html	2016
Aspergillus pulvericola CBS 137327	https://mycocosm.jgi.doe.gov/ Asppul1/Asppul1.home.html	2019
Aspergillus roseoglobulosus CBS 112800	https://mycocosm.jgi.doe.gov/ Aspros1/Aspros1.home.html	2015
Aspergillus sclerotiorum CBS 549.65	https://mycocosm.jgi.doe.gov/ Aspsc1/Aspsc1.home.html	2018
Aspergillus sesamicola CBS 137324	https://mycocosm.jgi.doe.gov/ Aspses1/Aspses1.home.html	2019
Aspergillus subramanianii CBS 138230	https://mycocosm.jgi.doe.gov/ Aspsubr1/Aspsubr1.home.html	2019
Aspergillus westlandensis CBS 123905	https://mycocosm.jgi.doe.gov/ Aspwes1/Aspwes1.home.html	2017
